# Supplementary material for: Minimal exposure durations reveal visual processing priorities for different stimulus attributes
Source: Nat Commun. 2024 Oct 2;15:8523. doi: 10.1038/s41467-024-52778-5 (PMC11447214; doi:10.1038/s41467-024-52778-5)
Supplement: Supplementary file 3 — Reporting Summary [file 41467_2024_52778_MOESM3_ESM.pdf]

nature portfolio

Corresponding author(s): Renzo Lanfranco
Last updated by author(s): Aug 19, 2024

Reporting Summary

Nature Portfolio wishes to improve the reproducibility of the work that we publish. This form provides structure for consistency and transparency in reporting. For further information on Nature Portfolio policies, see our Editorial Policies and the Editorial Policy Checklist.

Statistics

For all statistical analyses, confirm that the following items are present in the figure legend, table legend, main text, or Methods section.

n/a	Confirmed	
☐	☒	The exact sample size (*n*) for each experimental group/condition, given as a discrete number and unit of measurement
☐	☒	A statement on whether measurements were taken from distinct samples or whether the same sample was measured repeatedly
☐	☒	The statistical test(s) used AND whether they are one- or two-sided<br>*Only common tests should be described solely by name; describe more complex techniques in the Methods section.*
☐	☒	A description of all covariates tested
☐	☒	A description of any assumptions or corrections, such as tests of normality and adjustment for multiple comparisons
☐	☒	A full description of the statistical parameters including central tendency (e.g. means) or other basic estimates (e.g. regression coefficient) AND variation (e.g. standard deviation) or associated estimates of uncertainty (e.g. confidence intervals)
☐	☒	For null hypothesis testing, the test statistic (e.g. *F*, *t*, *r*) with confidence intervals, effect sizes, degrees of freedom and *P* value noted<br>*Give P values as exact values whenever suitable.*
☐	☒	For Bayesian analysis, information on the choice of priors and Markov chain Monte Carlo settings
☒	☐	For hierarchical and complex designs, identification of the appropriate level for tests and full reporting of outcomes
☐	☒	Estimates of effect sizes (e.g. Cohen's *d*, Pearson's *r*), indicating how they were calculated

Our web collection on statistics for biologists contains articles on many of the points above.

Software and code

Policy information about availability of computer code

Data collection	Stimulus presentation and data collection were performed using Psychtoolbox 3 (https://psychtoolbox.org/) using Matlab R2017b (The MathWorks, Inc).
Data analysis	Data were analysed using open-source tools developed for Matlab (R2021b, The MathWorks Inc), including EEGLAB, Brainstorm, the Amsterdam Decoding and Modeling (ADAM) toolbox, and custom code.

For manuscripts utilizing custom algorithms or software that are central to the research but not yet described in published literature, software must be made available to editors and reviewers. We strongly encourage code deposition in a community repository (e.g. GitHub). See the Nature Portfolio guidelines for submitting code & software for further information.

Data

Policy information about availability of data

All manuscripts must include a data availability statement. This statement should provide the following information, where applicable:

- Accession codes, unique identifiers, or web links for publicly available datasets
- A description of any restrictions on data availability
- For clinical datasets or third party data, please ensure that the statement adheres to our policy

Data and code are publicly available on the Open Science Framework: https://doi.org/10.17605/OSF.IO/9F2E8

Research involving human participants, their data, or biological material

Policy information about studies with human participants or human data. See also policy information about sex, gender (identity/presentation), and sexual orientation and race, ethnicity and racism.

Reporting on sex and gender	We collected gender information (self-reported) with the aim of ensuring gender-balanced samples for each experiment. However, since none of our research questions or hypotheses were focused on gender-related differences, we did not include this information in any of our analyses.
Reporting on race, ethnicity, or other socially relevant groupings	None of the variables defined in our study hold social relevance in this context.
Population characteristics	See Behavioural and social sciences section.
Recruitment	Participants were recruited through a Université libre de Bruxelles online platform for cognitive studies. Advertisement included information about the visual nature of the study, the length of the study, the equipment involved (LCD tachistoscope, EEG), compensation, and requirements to sign up (age range, no history of neurological or psychiatric disorders). No information about hypotheses or analyses was included. Before confirming their participation, they were contacted via email to confirm that they met the criteria.<br><br>Experiments 1-4 employed an LCD tachistoscope, a computer with MATLAB and a keyboard. Behavioural data were collected by this computer. Experiments 5-6 also employed a 64-electrode electroencephalography (EEG) system and a second laptop, which collected the EEG data.<br><br>The researcher was blinded to experimental conditions since these were administered by a computer code following predefined parameters. Blocks and trials were counterbalanced and randomised, respectively, based on a computer code. The researcher was not blinded to the study hypotheses. Only the researcher and the participant were in the laboratory room.
Ethics oversight	ULB Faculty Ethical Advisory Committee of the Faculty of Psychological Sciences and Education.

Note that full information on the approval of the study protocol must also be provided in the manuscript.

Field-specific reporting

Please select the one below that is the best fit for your research. If you are not sure, read the appropriate sections before making your selection.

☐ Life sciences ☒ Behavioural & social sciences ☐ Ecological, evolutionary & environmental sciences

For a reference copy of the document with all sections, see nature.com/documents/nr-reporting-summary-flat.pdf

Behavioural & social sciences study design

All studies must disclose on these points even when the disclosure is negative.

Study description	The six experiments included in this study were quantitative. Experiments featured a psychophysical component with a within-subject design, incorporating factors such as facial expression, emotional expression, and exposure durations. They collected detection and identification reports, as well as subjective awareness reports. Experiments 5 and 6, comprising the EEG component, also adopted a within-subject design and employed similar factors, except for Experiment 6, where stimulus category (face or object) and exposure duration served as the within-subject factors.
Research sample	Data were collected from 210 participants across 6 experiments (see the Methods section for details). Participants were recruited for the study through advertisements posted on a ULB online platform for human studies, which particularly targets established groups of university students in Belgium interested in participating in paid studies, as well as in university buildings. To be eligible to participate, volunteers needed to be between 18 and 40 years of age and have no history of neurological or psychiatric disorders. Regarding demographic information, we only collected self-reported data on age and gender.
Sampling strategy	Random sampling was conducted from volunteers who registered online. Participants with a particular interest in visual perception and EEG may have been more likely to volunteer (potential self-selection bias). However, we do not think this influenced our results.<br><br>Sample sizes were determined based on those of previous studies in the field: since most psychophysical studies typically recruit around 16 participants per experiment, and most EEG experiments recruit around 28 participants per experiment, we aimed for a target sample of 32 participants per experiment. For Experiments 5 and 6, an a priori power analysis was added (see Supplementary Note 10), which supported this target sample size.
Data collection	Data collection took place in a visual perception laboratory at the Centre for Research in Cognition and Neurosciences of the Université libre de Bruxelles (Belgium), employing a custom-made LCD tachistoscope, an EEG system, and a dedicated research laptop for control. Prior to the experiment, all participants were required to read and sign a consent form. In behavioural studies,

participants were briefed on the task they were to undertake and then given a 5-minute practice session before proceeding to the main task. For EEG studies, electrode placement occurred after participants signed the consent form but before they received instructions for the main task. The room was softly lit, and participants were instructed to place their chin on a chin rest and gaze at the main screen of the LCD tachistoscope through a viewing aperture.

Timing

The data collection for Experiments 1, 2, 5, and 6, as well as Control Experiments 1 and 2, took place between January and April 2019. Data collection for Experiments 3 and 4 occurred between September and November 2023.

Data exclusions

In Experiment 1, 35 participants were recruited, but 3 were subsequently excluded: 2 individuals failed to provide a response on more than 5% of the trials, and 1 displayed chance accuracy across all exposure durations, indicating a lack of engagement with the task. For Experiment 2, 34 participants were initially recruited, with 2 being excluded due to failure to respond on more than 5% of the trials. Similarly, in Experiment 3, 34 participants were recruited, but 2 were excluded for the same reason. In Experiment 4, 33 participants were recruited, and 1 was excluded because of a failure to provide a response on more than 5% of the trials. For Experiment 5, 36 participants were recruited, but 4 were excluded due to the presence of more than 15% noisy electrodes during the EEG pre-processing stage. Lastly, in Experiment 6, although 38 participants were recruited, 6 were excluded as they presented with more than 15% noisy electrodes during the pre-processing stage. These exclusion criteria were decided before data collection started.

Non-participation

No participants withdrew or declined participation.

Randomization

Each participant completed all conditions as the experiments in our study employed a within-subject design, thus avoiding the need to allocate participants into different groups. The experiments were conducted sequentially, with participants recruited for each experiment as it was being carried out.

Reporting for specific materials, systems and methods

We require information from authors about some types of materials, experimental systems and methods used in many studies. Here, indicate whether each material, system or method listed is relevant to your study. If you are not sure if a list item applies to your research, read the appropriate section before selecting a response.

Materials & experimental systems

n/a	Involved in the study
☒	☐ Antibodies
☒	☐ Eukaryotic cell lines
☒	☐ Palaeontology and archaeology
☒	☐ Animals and other organisms
☒	☐ Clinical data
☒	☐ Dual use research of concern
☒	☐ Plants

Methods

n/a	Involved in the study
☒	☐ ChIP-seq
☒	☐ Flow cytometry
☒	☐ MRI-based neuroimaging

Plants

Seed stocks

NA

Novel plant genotypes

NA

Authentication

NA
